# Epidemiology of *Clostridium difficile* infection in two tertiary-care hospitals in Perth, Western Australia: a cross-sectional study

**DOI:** 10.1002/nmi2.43

**Published:** 2014-04-01

**Authors:** N F Foster, D A Collins, S L Ditchburn, C N Duncan, J W van Schalkwyk, C L Golledge, A B R Keed, T V Riley

**Affiliations:** 1 School of Pathology and Laboratory Medicine, The University of Western Australia Perth, Western Australia, Australia; 2 Sir Charles Gairdner Hospital Perth, Western Australia, Australia; 3 Division of Microbiology and Infectious Diseases, PathWest Laboratory Medicine WA, Queen Elizabeth II Medical Centre Perth, Western Australia, Australia; 4 Department of Infectious Diseases and Microbiology, Royal Perth Hospital Perth, Western Australia, Australia

**Keywords:** Community-associated *Clostridium difficile* infection, healthcare facility-associated *Clostridium difficile* infection, molecular epidemiology, ribotype 244

## Abstract

The epidemiology of *Clostridium difficile* infection (CDI) has changed over time and between countries. It is therefore essential to monitor the characteristics of patients at risk of infection and the circulating strains to recognize local and global trends, and improve patient management. From December 2011 to May 2012 we conducted a prospective, observational epidemiological study of patients with laboratory-confirmed CDI at two tertiary teaching hospitals in Perth, Western Australia to determine CDI incidence and risk factors in an Australian setting. The incidence of CDI varied from 5.2 to 8.1 cases/10 000 occupied bed days (OBDs) at one hospital and from 3.9 to 16.3/10 000 OBDs at the second hospital. In total, 80 patients with laboratory-confirmed CDI met eligibility criteria and consented to be in the study. More than half (53.8%) had hospital-onset disease, 28.8% had community-onset and healthcare facility-associated disease and 7.5% were community-associated infections according to the definitions used. Severe CDI was observed in 40.0% of these cases but the 30-day mortality rate for all cases was only 2.5%. Besides a shorter length of stay among cases of community-onset CDI, no characteristics were identified that were significantly associated with community-onset or severe CDI. From 70 isolates, 34 different ribotypes were identified. The predominant ribotypes were 014 (24.3%), 020 (5.7%), 056 (5.7%) and 070 (5.7%). Whereas this study suggests that the characteristics of CDI cases in Australia are not markedly different from those in other developed countries, the increase in CDI rate observed emphasizes the importance of surveillance.

## Introduction

*Clostridium difficile* is an important nosocomial pathogen and the most frequently diagnosed cause of infectious hospital-acquired diarrhoea. *Clostridium difficile* infection (CDI) has a wide clinical spectrum, varying from asymptomatic carriage, to mild diarrhoea, to pseudomembranous colitis 1. *Clostridium difficile* produces two main toxins, toxin A and toxin B, which belong to the large clostridial toxin family. The genes for toxins A and B, *tcdA* and *tcdB*, respectively, are located on the chromosome in a 19.6-kb pathogenicity locus together with three accessory genes 2. Some strains also produce an actin-specific ADP-ribosyltransferase known as binary toxin (CDT) 3, the importance of which is still not clear. Although most clinically important strains produce both toxins, toxin A-negative, toxin B-positive (A^−^ B^+^) strains have been widely reported 4. In recent years, an increase in the frequency and severity of CDI has been associated with the emergence of a binary toxin-producing strain of *C. difficile* NAP1/027 (North American PFGE type 1, UK PCR ribotype 027) 5. This fluoroquinolone-resistant strain has been linked to epidemics in North America and Europe.

Major risk factors for clinically apparent CDI include antimicrobial therapy, hospitalization, residence in a long-term care facility, older age (≥65 years), and increased length of hospital stay 1. Little is known about the epidemiology of CDI in Australia. Given this lack of information, we undertook a study of CDI in two large teaching hospitals in Perth, Western Australia. The primary objectives were to estimate the incidence of CDI cases for hospitalized adult patients and to describe the profile of patients with the laboratory-confirmed infection. The prevalence of circulating ribotypes was also determined to understand the molecular epidemiology of CDI in Western Australia.

## Materials and Methods

### Setting and study design

This was a prospective, observational, epidemiological study conducted at the Sir Charles Gairdner Hospital (SCGH), a 600-bed tertiary teaching hospital, and the Royal Perth Hospital (RPH), an 855-bed tertiary teaching hospital, both located in Perth, Western Australia. Stool samples sent to the laboratory for *C. difficile* testing were monitored from December 2011 to May 2012. All adult patients who submitted loose stool samples for *C. difficile* testing over this period were considered for the study. A minimum target of 35 patients with laboratory-confirmed CDI per Western Australian hospital was set. The study was approved by the Sir Charles Gairdner Group (SCGG) and RPH Human Research Ethics Committees (SCGG Ref. 2011-133 and RPH Ref. RA-11/036). In Western Australia, next-of-kin/guardian/carer consent on behalf of the patient is not acceptable so patients who could not provide consent were not approached about the study.

### Definitions and collection of data

Recently recommended definitions were applied in this study 6,7. Patients were classified as having ‘laboratory-confirmed CDI’ if they experienced the passage of three or more unformed or loose stools conforming to the shape of a container (diarrhoea) within a 24-h period and had a *C. difficile*-positive laboratory test result. The laboratory testing method was Becton Dickinson GeneOhm™, which detects the toxin B gene (BD, Franklin Lakes, NJ, USA). Specimens from SCGH patients were screened using a glutamate dehydrogenase immunoassay (C. DIFF CHEK™-60; Alere, Sinnamon Park, Qld, Australia) before GeneOhm analysis. All specimens that were glutamate dehydrogenase-negative were regarded as negative for *C. difficile*. The consenting patient's hospital record was reviewed to confirm the CDI episode. Other data regarding patient location at onset, risk factors and outcomes were abstracted from the medical record and by interviewing the patient using a standardized questionnaire. The number of occupied bed days (OBDs) was extracted from the respective hospital administrative databases. Patients fulfilling the case definition of a laboratory-confirmed CDI were further classified as having: (1) ‘community-onset CDI’ (when they experienced diarrhoea in the community or 48 h or less after admission to the hospital) or ‘hospital-onset CDI’ (when the onset of diarrhoea was more than 48 h after admission to a hospital); and (2) ‘healthcare facility (HCF) -associated CDI’ (when they had been discharged from a healthcare facility within the previous 4 weeks from the onset of CDI symptoms), ‘community-associated CDI’ (when they had not been discharged from a healthcare facility within the previous 12 weeks from the onset of CDI symptoms) or ‘indeterminate CDI’ (when they had been discharged from a healthcare facility within the previous 4–12 weeks from the onset of CDI symptoms). Severe CDI in a patient was defined as one or more of the following: leucocytosis with a white blood cell count of >15 000 cells/μL, a serum creatinine level >1.5 times the pre-morbid level (or if the pre-morbid level was unknown, 1.5 times above the normal range), evidence of severe colitis (abdominal or radiological signs), temperature >38.5°C, admission to an intensive care unit for complications associated with CDI, surgery (e.g. colectomy) for treatment of toxic megacolon, perforation or refractory colitis due to CDI, death within 30 days due to or related to CDI.

### *Clostridium difficile* culture and ribotyping

The presence of viable *C. difficile* was confirmed by culture. Direct culture of the specimen was performed on CCFA (cycloserine-cefoxitin-fructose agar containing 0.1% taurocholate; PathWest Laboratory Medicine Excel Media 8). Broth enrichment in Robertson's cooked meat medium containing 5 mg/L of gentamicin, 250 mg/L of cycloserine and 8 mg/L of cefoxitin was performed concurrently and an ethanol-shocked aliquot was subcultured on CCFA 9,10. The identity of putative *C. difficile* colonies was confirmed by morphology, odour and chartreuse fluorescence on blood agar, and l-proline aminopeptidase DIATABS™ (Rosco Diagnostica, Taastrup, Denmark) reaction.

The PCR ribotyping was performed as previously described 11; PCR ribotyping reaction products were concentrated using the Qiagen MinElute PCR Purification kit (QIAGEN, Venlo, Limburg, the Netherlands) and resolved on the QIAxcel capillary electrophoresis platform (QIAGEN). Cluster analysis of PCR ribotyping band profiles was performed using the Dice similarity coefficient with relationships represented in a UPGMA dendrogram within BioNumerics™ software package v.6.5 (Applied Maths, Saint-Martens-Latem, Belgium). Ribotypes were identified by comparison of the band profiles with our reference library, which consisted of a collection of 50 UK ribotypes that included 15 reference strains from the European Centre for Disease Prevention and Control and the most prevalent PCR ribotypes currently circulating in Australia (B. Elliott, T. V. Riley *et al*., unpublished data). Isolates that could not be identified with the available reference library were designated with internal (QX) nomenclature.

### Statistical methods

Graphpad Instat V3.06 was used to calculate prevalence ratios and their 95% confidence intervals, and to perform Fisher's exact and chi-square analyses. A value of p ≤0.05 was considered statistically significant.

## Results

**Figure 1:**
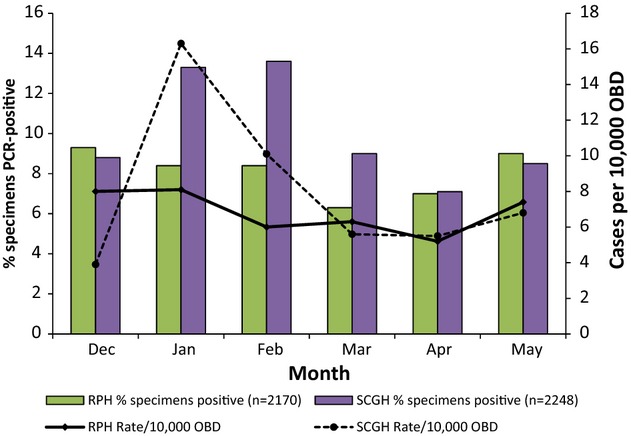
Rates of *Clostridium difficile* infection (CDI) and proportion of specimens positive for *C. difficile* at Royal Perth Hospital and Sir Charles Gairdner Hospital during the study period, by month.

A total of 2170 specimens from RPH and 2248 specimens from SCGH were tested for *C. difficile* over the study period (Fig.1). At RPH, the proportion of tests that were PCR-positive varied from a low of 6.3% in March to a high of 9.0% in May, with an average of 8.1% positive. At SCGH, the proportion of PCR-positive tests varied from a high of 13.6% in February to a low of 7.1% in April, with an average of 10.1% positive. The difference in proportion of tests positive between RPH and SCGH was statistically significant in February 2012 (chi-squared p <0.0389).

The incidence of CDI was estimated from the number of new patients with *C. difficile* testing requested who had stool specimens that were positive for *C. difficile*. At RPH and SCGH throughout the study period this reflected the proportion of specimens tested that were positive for *C. difficile* (Fig.1). There was little variation at RPH during the nearly 6-month study period with rates varying from 5.2 to 8.1 cases/10 000 OBDs. However, at SCGH, the rates fluctuated from a low of 3.9/10 000 OBDs in December 2011 to a high of 16.3/10 000 OBDs in January 2012 before dropping to rates similar to RPH for March, April and May 2012. The average rate at RPH for the study period was 6.8 cases/10 000 OBDs while the average rate at SCGH was 8.0 cases/10 000 OBDs.

**Figure 2:**
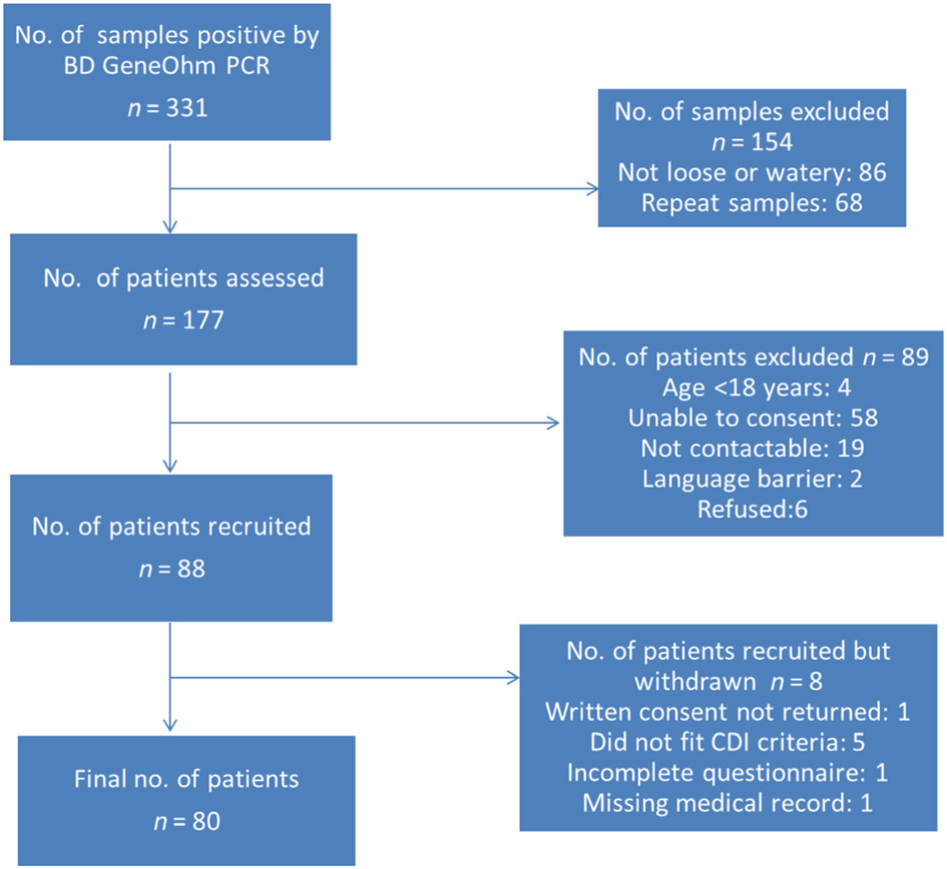
Recruitment strategy.

From 331 PCR-positive stool specimens from patients at RPH and SCGH, 80 cases were recruited for further study (Fig.2). Initially 154 were excluded because the stools were not loose or watery (*n* = 86) or they were repeat samples from previously PCR-positive patients (68). From the remaining 177 patients for possible inclusion in the study, a total of 88 patients were recruited, with the main reason for exclusion being inability to consent (58 patients, 32.7%). Of the 88 patients recruited, eight were withdrawn, the main reason being that they did not meet the definition of CDI (five patients). Of eligible patients, only six declined to participate in the study, giving a response rate of 93.6%.

The characteristics of the 80 patients with CDI are shown in Table1. The median age of the patients was 60.5 years of age and just over half (51.3%) were male. Overall, 26.3% of people with CDI had three or more co-morbidities, with the most common being: oesophageal reflux (46.3%), hypertension (42.5%), chronic pulmonary disease (36.3%), chronic renal disease (36%), cancer (35%), immune deficiency (35%) and rheumatological disease (31.3%). A history of CDI was noted in 17.5% of patients and 13.8% of patients had undergone a colonoscopy, sigmoidoscopy or oesophagogastroduodenoscopy during the admission. The median length of stay for patients who acquired CDI was 15 days compared with a median length of stay of 6.0 and 6.1 days for patients at RPH and SCGH, respectively, during the same period.

**Table 1:** Characteristics of *Clostridium difficile* infection cases

Characteristic	Number of cases (%) (*n* = 80)
Age (years)	
Median	60.5
Interquartile range	49–71
Male	41 (51.3)
Ethnicity	
Caucasian	76 (95.0)
Aboriginal	3 (4.8)
Asian	1 (1.3)
Significant past medical history	
Oesophageal reflux	37 (46.3)
Hypertension	34 (42.5)
Chronic pulmonary disease	32 (40.0)
Chronic renal disease	29 (36.3)
Cancer	28 (35.0)
Immune deficiency	28 (35.0)
Rheumatological disease	25 (31.3)
Chronic liver disease	22 (27.5)
Heart disease	19 (23.8)
Gastritis	16 (20.0)
Type 2 diabetes mellitus	13 (16.3)
Colitis	10 (12.5)
Gastric ulcer	8 (10.0)
Connective tissue disorder	8 (10.0)
Irritable bowel disease	8 (10.0)
Diverticulitis	7 (8.8)
Cerebrovascular disease	2 (2.5)
Type 1 diabetes	1 (1.3)
History of CDI	14 (17.5)
Previous antibiotic use in the past 3 months	50 (62.5)
Amoxicillin/amoxicillin clavulanate	10 (12.5)
Cephalosporins	9 (11.3)
Metronidazole	6 (7.5)
Piperacillin tazobactam	5 (6.3)
Vancomycin	4 (5.0)
Clindamycin	4 (5.0)
Trimethoprim sulfamethoxazole	2 (2.5)
Fluoroquinolones	1 (1.3)
Others	27 (33.8)
Duration of previous antibiotic use (days)	
Median	14
Interquartile range	8–49
Antibiotic use this admission (before specimen collection)	42 (52.5)
Piperacillin tazobactam	7 (8.8)
Amoxicillin clavulanate	5 (6.3)
Meropenem	5 (6.3)
Vancomycin	4 (5.0)
Fluoroquinolones	4 (5.0)
Ciprofloxacin	3 (3.8)
Moxifloxacin	1 (1.3)
Cephazolin	3 (3.8)
Metronidazole	1 (1.3)
Fluconazole	1 (1.3)
Ceftriaxone	1 (1.3)
Cephalexin	1 (1.3)
Received enteral feeding	1 (1.3)
Underwent OGD/colonoscopy/sigmoidoscopy	11 (13.8)
OGD	7 (8.8)
Colonoscopy	3 (3.8)
Sigmoidoscopy	2 (2.5)
Number of stools in the last 24 h	
Median	5.5
Interquartile range	4–10
LOS (days)	
Median	15
Interquartile range	7.5–40

CDI, *Clostridium difficile* infection; OGD, oesophagogastroduodenoscopy; LOS, length of stay.

Most patients (62.5%) recalled taking antibiotics within the preceding 3 months (Table1). The most commonly consumed antibiotics preceding CDI were amoxicillin / amoxicillin clavulanate (12.5%) and cephalosporins (11.3%). Just over half (52.5%) of the CDI patients had consumed antibiotics during the admission when CDI was diagnosed. The most common antibiotics received for these patients were: piperacillin tazobactam (8.8% of patients in the study had previous exposure), cephalosporins (6.3%), amoxicillin clavulanate (6.3%) and meropenem (6.3%). Vancomycin, commonly used in the treatment of CDI, was also implicated in four patients (5%) before the onset of diarrhoea.

Metronidazole predominated as the treatment of choice for CDI with 58.8% of patients receiving solely metronidazole and 26.3% receiving both metronidazole and vancomycin (Table1). Interestingly, only five patients (6.3%) received vancomycin alone despite a number of severe cases.

A total of 32 patients (40.0%) in the study had severe CDI as defined, however, only two patients recruited into the study (2.5%) died within 30 days. The 90-day crude mortality rate was 16.3%; six of the 13 deaths were among patients with severe CDI. A comparison of clinical signs of CDI in patients with severe versus non-severe disease is given in Table2.

**Table 2:** Clinical signs of *Clostridium difficile* infection

Clinical sign	All CDI (%)	Non-severe CDI (%) (*n* = 48)	Severe CDI (%) (*n* = 32)	Prevalence ratio (95% CI)
Dehydration	53 (66.3)	31 (64.6)	22 (68.8)	1.07 (0.78–1.46)
Hypotension	14 (17.5)	7 (14.6)	7 (21.9)	1.50 (0.58–3.87)
Ileus	2 (2.5)	0	2 (6.3)	
Loss of appetite	68 (85.0)	42 (87.5)	26 (81.3)	0.93 (0.76–1.13)
Malaise	49 (61.3)	27 (56.3)	22 (68.8)	1.22 (0.87–1.72)
Megacolon	1 (1.3)	0	1 (3.1)	
Nausea	35 (43.8)	18 (37.5)	17 (53.1)	1.42 (0.87–2.31)
Weight loss	48 (60.0)	29 (60.4)	19 (59.4)	0.98 (0.68–1.42)

CDI, *Clostridium difficile* infection.

Of the 80 CDI cases 55% were readmissions, defined as having a previous hospital admission for any reason within the last 4 weeks. The median number of admissions in the last 3 months was 1 (interquartile range, 0–2.3). More than half the CDI cases (53.8%) were hospital-onset and 32.6% of these met the definition of severe CDI. Of the community-onset cases (46.3%), 23 (62.2%) were HCF-associated, 6 (16.2%) were community-associated, and 8 (21.6%) were of indeterminate nature as defined. A comparison between hospital and community-onset CDI is given in Table3. There were no statistically significant differences in risk factors or outcomes examined other than length of stay where patients with community-onset CDI stayed for significantly less time than patients with hospital-onset CDI. Interestingly, severe CDI was more common among community-onset cases but this did not reach statistical significance (Fisher's exact p 0.173).

**Table 3:** A comparison of hospital-onset and community-onset *Clostridium difficile* infection

Risk factor	Hospital-onset CDI (%) (*n* = 43)	Community-onset CDI (%) (*n* = 37)	Prevalence ratio (95% CI)
Age ≥65 years	17 (39.5)	15 (40.5)	1.03 (0.6–1.76)
Male	20 (46.5)	21 (56.8)	1.22 (0.8–1.87)
Two or more chronic diseases	25 (58.1)	15 (40.5)	0.70 (0.48–1.11)
History of CDI	6 (14.0)	8 (21.6)	1.55 (0.6–4.06)
Severe CDI	14 (32.6)	18 (48.6)	1.49 (0.87–2.57)
Previous antibiotic use in the past 3 months	25 (58.1)	25 (67.6)	1.16 (0.83–1.63)
Antibiotic use this admission (before specimen collection)	26 (60.5)	16 (43.2)	0.72 (0.46–1.11)
Readmission	21 (48.8)	23 (62.2)	1.27 (0.86–1.89)
LOS ≥20 days	26 (60.5)	8 (21.6)	0.36 (0.19–0.69)

CDI, *Clostridium difficile* infection; LOS, length of stay.

**Figure 3:**
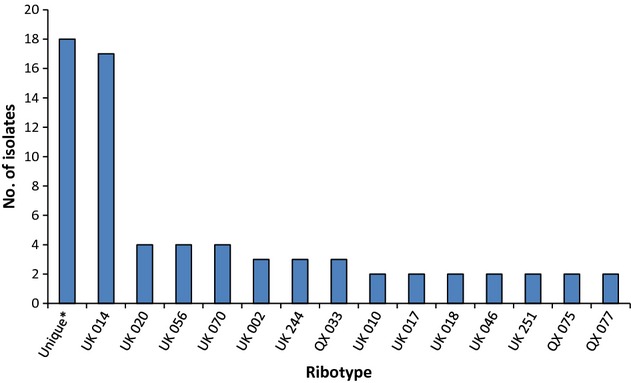
Ribotypes of *Clostridium difficile* isolated from study participants at Royal Perth Hospital and Sir Charles Gairdner Hospital during the study period. Unique ribotypes (*) included 001, 015, 054, 087, 103, 237, 297 and 014/020-group, and local ribotypes QX 001, 013, 024, 050, 081, 097, 103, 113, 161 and 227.

Of the 80 cases of laboratory-confirmed CDI, *C. difficile* was isolated from 70, 34 at RPH and 36 at SCGH. The ribotypes of these 70 isolates are shown in Fig.3. Of the 32 ribotypes present, the most common was ribotype 014 accounting for one-quarter (24.3%) of isolates. The next most common were ribotypes 020, 056 and 070 each at 5.7%, followed by ribotypes 002, 244 and QX 033 (103-like), all at 4.3%. Eighteen (25.7%) ribotypes were unique, of which 11 could not be assigned a UK ribotype from our reference collection.

## Discussion

Little is known about the epidemiology of CDI in Australia. A 10-year study at SCGH in Western Australia between 1983 and 1992, showed that the incidence of CDI increased from ∼3 cases per 10 000 OBDs in 1983 to nearly 7 cases per 10 000 OBDs in 1988 before stabilizing 12. A significant decrease in the incidence of CDI at SCGH was observed 10 years later in 1998 when the use of third-generation cephalosporins was restricted within the hospital, and rates declined to <2/10 000 OBDs 13. Recently, routine surveillance of CDI commenced in Victorian public hospitals and a rate of CDI of 2.2/10 000 OBDs was reported for October 2010 to March 2011 14. The rates seen in the current study were similar to those seen at SCGH in the late 1980s and exceeded those recently reported in Victoria. The average rate at RPH for the study period was 6.8 cases/10 000 OBDs while the average rate at SCGH was 8.0 cases/10 000 OBDs (Table1). These rates are similar to those reported for the Province of Ontario (8.1/10 000 patient days) in Canada in a study performed from November 2004 until April 2005 but less than the rates reported for Quebec Province in the same study (13/10 000 patient days) 15. It is interesting to note however that the rate at SCGH in January 2012 (16.3/10 000 OBDs) exceeded this latter figure. This very high rate could not be explained by any obvious clustering of cases in certain wards or by an excess of particular strains, and requires further investigation. Differences between rates at RPH and SCGH were also reflected in the higher proportion of samples that were positive at SCGH (Fig.1), so may be due to a difference in thresholds for patient testing. Molton *et al*. 16 reported a similar increase in Singapore. CDI rates increased three-fold, from 4.2 per 10 000 patient days to 12.1 per 10 000 patient days from March 2010 to April 2012.

Community-acquired CDI has been investigated in Western Australia previously. In a study of diarrhoeal disease with community acquisition and onset, *C. difficile* was the second most commonly detected pathogen after *Campylobacter* species 17. In the present study, 28.8% of the CDI cases were community-onset, HCF-associated, 7.5% were community-associated and 10.0% were of indeterminate nature as defined. A similar breakdown of cases was reported from the Victorian public hospital surveillance system where the same criteria were used for the time/place of onset of cases 14. This is likely to be an under-representation of community-acquired CDI. The definitions used for this study 6 mean that any patient who develops CDI within 4 weeks of discharge from an HCF is classified as having HCF-associated, community-onset disease, and between 4 and 12 weeks the attribution was indeterminate. However, many of these cases will be true community-acquired infection given that there is now good evidence that the rates of community-acquired CDI around the world are increasing 18,19. It was noteworthy that 19% of cases were haematology/oncology patients who, because of their frequent visits to healthcare facilities, are always classified as having HCF-associated CDI (data not shown). However, many of these patients are likely to have acquired their infection in the community.

In Australia, CDI has been driven by exposure to cephalosporins 13. Not surprisingly, in the present study, previous exposure to broad-spectrum antibiotics (piperacillin tazobactam, amoxicillin clavulanate, cephalosporins and carbapenems) was again associated with CDI. This association is similar to other studies worldwide where cephalosporins as well as other broad-spectrum antibiotics are the most frequently implicated antibiotics 13,20,21.

Antibiotic use in the preceding 3 months was the only significant risk factor identified by Leonard *et al*. 22 in one of only two Australian studies assessing risk factors for CDI. In this small case–control study of diarrhoeal patients at RPH from 2009–2010, the crude mortality rate (12%) was similar to the 90-day mortality rate observed in the present study. In Tasmania in 1997, severe underlying disease, renal impairment, exposure to antineoplastic agents, and the use of total parenteral nutrition or nasogastric feeding, all well-known risk factors for CDI 20, also increased the risk of developing CDI 23. The crude mortality rate was 17.2%, and factors associated with a poor prognosis were older age, severe underlying disease, renal impairment and failure to treat with metronidazole or vancomycin. It would appear that little has changed in Australia in relation to risk factors.

Although over a third of cases were considered severe, the 30-day mortality rate in the present study (2.5%) was low compared with the 30-day attributable mortalities in studies from North America: 6.9% overall in Quebec, Canada, during a ribotype 027 outbreak 24. This may reflect the lack of ribotype 027. However, as noted in Fig.2, a third of patients assessed were not capable of consenting to inclusion in the study. In many instances this was because they were too ill and therefore it is possible that the mortality rate could be higher.

As part of a study that evaluated a new treatment for CDI, Cheknis *et al*. 25 typed 24 Australian *C. difficile* isolates recovered in the mid-2000s by restriction endonuclease analysis and found that two-thirds of them were uncommon types (compared with isolates from North America and Europe). A quarter of the isolates belonged to restriction endonuclease analysis group Y, a group that corresponds to PCR ribotypes 014 and 020. PCR ribotyping is a widely used and highly discriminatory method for investigating intra-species variation among *C. difficile* isolates. In the present study, 32 different ribotypes were identified among 70 isolates from consecutive patients, emphasizing the diversity of strains in Australia. Ribotype 014 was again the most commonly detected at a prevalence of 24.3%. This ribotype seems well adapted to surviving in the hospital environment as it was common in the UK before the emergence of the ribotype 027 epidemic strain 11 and is still common in Europe (∼10%) 26,27. The study by Stubbs *et al*. 11 from the UK Anaerobe Reference Unit of 2030 *C. difficile* strains distinguished 116 types while a study of 330 isolates in a Swedish county distinguished 53 types 28.

Besides ribotype 014 (which is often grouped with 020 due to difficulty distinguishing the two), the most prevalent ribotypes in Europe appear to be 001 (∼9%), 078 (∼8%), 018 (∼6%) and 106 (∼5%). A review of ribotyping results across Asia suggested that 017, 018, 014, 002 and 001 were the most prevalent in the region 29. In North America, ribotype 027 predominates (∼29%); other common ribotypes include 014 or 020 (∼12%), 002 (∼5%), 053 (∼5%) and 106 (∼5%) 30,31. With such a high prevalence of 014 and 020 in the present study, other ribotypes were generally less common than elsewhere with the exception of ribotypes 056 and 070 (both 6%). Ribotype 056 has been associated with more complicated infections 27.

Recently, *C. difficile* ribotype 027 was isolated for the first time in Australia from a patient who had returned from the USA 32 and, early in 2010, the first cluster of ribotype 027 cases was detected in Melbourne, Victoria 33. Interestingly, *C. difficile* ribotype 027 does not appear to have established in Australia and none was detected in the present study. It is possible that antibiotic prescribing practices in Australia have not favoured the emergence of this epidemic strain as the use of later fluoroquinolones is quite restricted. It is also possible that the geographic isolation of Australia is also responsible for the delayed appearance of ribotype 027. However, another potentially epidemic ribotype that produces binary toxin, ribotype 244, was detected at a prevalence of 4.3%. Although this appears low, it was the equal third most common ribotype found, and one that was not in Australia 2 years ago. Preliminary data from other States of Australia, and New Zealand, suggest that ribotype 244 infection may be associated with more severe disease 34. Also of interest, ribotype 237 (A^−^ B^+^ CDT^+^), a recently described strain from Australian livestock 35, was identified in one patient. Ribotype 078, the predominant livestock strain, which is associated with community-acquired infection in the northern hemisphere 19, was not seen. This strain was absent in recent Australian livestock surveys 36,37.

This study has highlighted a significant increase in the rate of CDI in Western Australia. Surveillance data from other Australian States suggest that similar increases have occurred Australia-wide, and that this may be at least partly due to community-acquired CDI 38,39. The contribution of community-acquired CDI to overall rates may also be masked by the current definitions used. The emergence of at least one new virulent strain of *C. difficile* is a concern, and this needs to be monitored. Risk factors for acquiring *C. difficile* in hospital appear to be unchanged; however, risk factors for community acquisition require further investigation.
